# Imbalance in MICOS Proteins in Rat Liver Mitochondria in an Induced Hyperthyroidism Model

**DOI:** 10.3390/cells14231877

**Published:** 2025-11-27

**Authors:** Natalya Venediktova, Ilya Solomadin, Anna Nikiforova, Tatiana Bessonova

**Affiliations:** 1 Institute of Theoretical and Experimental Biophysics of RAS, 142290 Pushchino, Russia; iliusmaster@rambler.ru (I.S.); nikiforanna@yandex.ru (A.N.); 2 Institute of Cell Biophysics of RAS (FRC PSCBR RAS), 142290 Pushchino, Russia; tatianabessonova66@gmail.com; 3 Vavilov Institute of General Genetics of RAS, 119991 Moscow, Russia

**Keywords:** cristae membranes, hyperthyroidism, MICOS, OPA1, cardiolipin, oxidative phosphorylation, calcium retention capacity

## Abstract

This study investigated rearrangements in the cristae structure and the possible relationship between these changes and the MICOS levels in the liver mitochondria of rats with experimentally induced hyperthyroidism. In hyperthyroid rats (HRs), the number, area, and perimeter of mitochondria were increased, and organelles of a worm-shaped, branched, highly elongated, or spherical shape appeared. A structural change in the mitochondria of HR liver was detected, consisting of a decrease in the number of cristae relative to the cross-section of the organelle. In some mitochondria, multilamellar bodies were detected. Hyperthyroidism caused an increase in the expression of genes and the level of proteins of the MIC60 subcomplex, with an unchanged level of the MIC10 subcomplex. Moreover, the levels of Sam50 and OPA1 in HRs were reduced. A functional assessment of HR mitochondria revealed changes in oxygen consumption, a decrease in membrane potential, and disruption of Ca^2+^ homeostasis. These data indicate that excess thyroid hormones cause partial changes in liver mitochondrial structure and an imbalance in the levels of Mic60 and Mic10 subcomplex proteins. The decreased levels of Sam50 and OPA1 proteins suggest their potential as targets for correcting mitochondrial dysfunction in metabolic disorders.

## 1. Introduction

In recent decades, there has been a significant increase in the number of patients worldwide with diseases associated with mitochondrial dysfunction [1,2,3]. Mitochondria play a key role in ATP production, regulation of redox balance, and apoptosis. Often, under the influence of genetic, environmental, and metabolic factors, the ability of mitochondria to energetically adapt is reduced, leading to chronic bioenergetic defects and cumulative cell damage [1,2,3]. An example of such a condition is hyperthyroidism, a pathology characterized by excessive production of thyroid hormones—triiodothyronine (T_3_) and thyroxine (T_4_). Normally, these hormones perform vital regulatory functions, exerting a complex effect on virtually all body systems. In hyperthyroidism (HT), the body is forced to adapt to increased energy needs by intensifying cellular respiration. This may lead to pronounced functional and structural changes in mitochondria. Mitochondrial dysfunction is known to be an important link in both the development and progression of HT, in particular due to the development of oxidative stress, changes in cellular respiration, and improper functioning of the mitochondrial quality control system and mitophagy [4,5,6]. High efficiency of energy metabolism is ensured by the unique structure of mitochondria, including the cristae of the inner membrane [7]. Three membrane-forming factors, namely dimeric F_1_Fo-ATP synthase, mitochondrial contact site and cristae organizing system (MICOS), and GTPase 1 (OPA1), have been shown to play a crucial role in the biogenesis and maintenance of cristae. Dimerization of ATP synthase is necessary for the formation of dimers of this protein, which bend the membrane, facilitating the formation of cristae. Mutations in the genes encoding ATP synthase subunits (e.g., ATP5ME) lead to cristae disruption and decreased efficiency of oxidative phosphorylation [8]. MICOS proteins stabilize the contact zones of the inner and outer membranes and regulate cristae morphology. The MICOS complex participates in the formation of crista junctions (CJs)—narrow connections between the cristae membrane (CM) and the inner boundary membrane (IBM). Dysfunction of the MICOS complex is associated with various mitochondrial pathologies. The OPA1 protein is involved in the fusion and fission of the inner membrane, maintaining its dynamics. In addition, OPA1 prevents the release of cytochrome c, suppressing uncontrolled apoptosis. Mutations in the OPA1 gene lead to autosomal dominant optic neuropathy and other mitochondrial pathologies [9]. Although disruption of mitochondrial cristae structure is observed in many human diseases, it remains unclear whether these changes are the primary cause or simply a contributing factor to disease progression. The aim of this study was to investigate changes in the structure of cristae and MICOS proteins in rat liver mitochondria using a model of experimentally induced hyperthyroidism. The development of methods for correcting mitochondrial architecture represents a promising therapeutic approach for a wide range of diseases. The results obtained in this study may not only provide new insights into mitochondrial functioning but also suggest directions for the development of therapeutic strategies for correcting this pathology and a number of other diseases associated with altered mitochondrial metabolism.

## 2. Materials and Methods

### 2.1. Experimental Model

Adult male Wistar rats (weight, 215–235 g; age, 3–3.5 months) were housed under controlled conditions (room temperature, constant humidity, 12 h:12 h dark–light cycle), food and water were provided ad lib. The animals were divided into 2 groups: control group (C) (*n* = 10) and rats injected with a solution of T_4_ (HT) (*n* = 10). Hyperthyroidism was simulated by intraperitoneal injection of thyroxine at a dose of 200 μg per 100 g of animal weight for 7 days [5]. Control rats (CRs) were injected with an equal volume of saline solution. At the end of the treatment period all rats were sacrificed. The serum was collected for analysis of free T_3_ and T_4_, lactate dehydrogenase, alanine aminotransferase and aspartate aminotransferase levels using the appropriate reagent kits (Vector-Best, Novosibirsk, Russia).

### 2.2. Total Carbonyl Colorimetric Assay

A Total Carbonyl Colorimetric Assay kit (E-BC-K171-M, Elabscience, Wuhan, China) was used to determine the amount of total carbonyl groups. Carbonyl can react with 2,4-dinitrophenylhydrazine and produce a kind of reddish brown hydrazone compounds, which has a specific absorbance peak at 370 nm. The content of carbonyl can be calculated according to the absorbance value.

### 2.3. Isolation of Rat Liver Mitochondria

The liver was cooled in an isolation medium, washed to remove blood, and then mechanically disrupted by pressing through a 1 mm diameter sieve. The tissue was subsequently homogenized in a Potter glass homogenizer with a Teflon pestle using a 1:10 (*w*/*v*) ratio of tissue to isolation medium. The isolation medium composition was as follows: 210 mM mannitol, 70 mM sucrose, 10 mM HEPES-KOH, 0.1% fatty acid-free BSA, and 0.5 mM EGTA (pH 7.4). The homogenate was subjected to a series of centrifugation steps: first at 700× *g* for 10 min to remove debris and nuclei, and then the supernatant was centrifuged at 5000× *g* for 10 min to sediment the mitochondria. The mitochondrial pellet was washed in the same medium without BSA and EGTA (medium B), and centrifuged again at 5000× *g* for 10 min. The final mitochondrial pellet was resuspended in Medium B at a concentration of 0.1 mL per gram of original liver tissue. Mitochondrial protein concentration was determined using the Lowry method [10]. All procedures involving mitochondrial isolation and subsequent handling were performed either in a cold room or on ice.

### 2.4. Assay of the Expression of MICOS Genes Using Quantitative Real-Time PCR

#### 2.4.1. Extraction of RNA

Isolation of total RNA from liver was performed using a total RNA isolation reagent, the ExtractRNA reagent (BC032, Evrogen, Moscow, Russia), containing a solution of phenol and guanidine isothiocyanate.

1 mL of ExtractRNA reagent was added to 50–100 mg of liver tissue crushed under liquid nitrogen, then total RNA was isolated according to the manufacturer’s protocol. The quality of RNA isolation was assessed using electrophoresis on a 1% agarose gel, as well as by measuring absorbance at 260 nm with a spectrophotometer. The purity of the RNA was determined by calculating the ratios of absorbance values at 260/280 and 260/230. To eliminate contamination from genomic DNA, the RNA samples were treated with DNase I (M0303, New England Biolabs, Ipswich, MA, USA) at 37 °C for 1 h. The enzyme was then inactivated by adding 50 mM EDTA to the mixture and heating it to 70 °C for 10 min. The concentration of extracted RNA was determined by a NanoDrop spectrophotometer. The reverse transcription reaction was performed following the provided protocol, utilizing a first-strand cDNA synthesis kit (SK022 Evrogen, Moscow, Russia) that includes the mouse leukemia virus reverse transcriptase (MMLV). The total RNA concentration in the reaction mixture was 2 μg.

#### 2.4.2. Quantitative Real-Time Polymerase Chain Reaction (RT-qPCR)

The reaction system was prepared in a 20 μL PCR tube composed of 4 μL of qPCRmix-HS SYBR (PK147, Evrogen, Moscow, Russia), 0.8 μL (0.4 μM) of the primer solution, 14.2 μL of water (RNase-free), and 1 μL of cDNA. A QuantStudio 1 amplifier (ThermoFisher Scientific, Waltham, MA, USA) was used for amplification. The sequences of the used primers are presented in Table 1. All the sequences were designed based on the analysis of the nucleotide sequences of the existing gene isoforms and were specific for the rat with NCBI Primer-BLAST software (https://www.ncbi.nlm.nih.gov/tools/primer-blast/index.cgi?GROUP_TARGET=on, accessed on 1 December 2022)). The amplification process consisted of the initial 3 min denaturation at 95 °C, 35 cycles of 15 s denaturation at 95 °C, 30 s annealing at 56 °C, and a 25-s extension step at 72 °C. After amplification, the melting stage was carried out for each amplified product. The first part of the melting process involved a brief denaturation of samples at 95 °C for 15 s and rapid annealing. Following this, the temperature was again slowly increased (0.6 °C/s) and the melting curve data were obtained from 56 °C (30 s) to 95 °C (15 s). The data were analyzed with the RT^2^ Profiler PCR Data Analysis (https://dataanalysis2.qiagen.com/pcr/ (accessed on 5 March 2022)). Gene expressions were normalized to housekeeping genes ribosomal protein lateral stalk subunit P2 (*Rplp2*) and peptidylprolyl isomerase A (*PPIA*). The changes in the levels of mRNA expressions of the studied genes, in HRs and CRs, were determined by the Livak’s method [11]. Fold change (2^−ΔΔCt^) is the normalized gene expression (2^−ΔCt^) in the HT group divided the normalized gene expression (2^−ΔCt^) in the control group, where ∆Ct = Ct (investigated gene of rats of the HT group or control group)—Ct (housekeeping gene of rats of the HT group or control group).

### 2.5. Sample Preparation, Electrophoresis and Immunoblotting Analysis

Western blotting analyses were performed as previously described [12]. Briefly, samples for electrophoresis were prepared from rat liver mitochondria or rat liver tissue by using RIPA buffer (Abcam, Cambridge, UK) with inhibitors cocktail (P8340, Sigma-Aldrich, St. Louis, MO, USA and ab201113 Abcam, Cambridge, UK). Liver tissue (10–15 mg) was homogenized in a special tube filled with beads (Precellys Lysing kit, Montigny-le-Bretonneux, France) for 20 s at 5000 rpm on ice. Then, samples were centrifuged at 12,100× *g* for 20 min, and the supernatant was stored at –80 °C. Electrophoresis and immunoblotting were performed using a standard technique according to [13]. The samples were separated by 12.5% or 15% SDS-PAGE and transferred to a 0.45 µm nitrocellulose membrane. We visualized the proteins of interest and relevant loading controls (Table 2) with ClarityWestern ECL substrate (Bio-Rad, Hercules, CA, USA). Imaging was performed on a Chemidoc Touch system, and the chemiluminescence was analyzed with ImageLab 5.2 software.

All primary antibodies were diluted 1:1000. The anti-GAPDH and VDAC were used as a loading control and were diluted 1:2000.

### 2.6. Electron Microscopy

Liver tissue samples (*n* = 3 for each group) were collected for electron microscopy within 1–1.5 min post-extraction. Selected specimens were fixed in primary fixative (2.5% glutaraldehyde in 0.1 M sodium cacodylate buffer, pH 7.4) for 4 h at 4 °C, then post-fixed in 1% osmium tetroxide (OsO_4_) for 3 h. After fixation, samples were dehydrated through a graded ethanol series (40% to 100%) followed by absolute acetone, and embedded in epoxy resin (Epon 812). Finally, 60–70 nm thick sections (Leica Microsystems, Wetzlar, Germany) were obtained and stained with 2% uranyl acetate and lead citrate. Electron microscopy was performed using a JEM-1400 transmission electron microscope (JEOL, Tokyo, Japan). A standard measurement method was used, which involves the manual contouring of mitochondrial cross sections. For 2D morphometric analysis, 6000×, 10,000× and 20,000× images were obtained and analyzed using Fiji open source software (1.52n) (https://fiji.sc/ (accessed on 2 October 2025)). Images at 6000× were used to measure the number, area and perimeter of mitochondria. Images at 20,000× were used for analysis of the number and width of lamellar regular cristae.

### 2.7. High-Resolution Respirometry

Mitochondrial functional analysis was conducted using the Oxygraph-2k high-resolution respirometry system (Oroboros Instruments, Innsbruck, Austria), featuring dual 2 mL chambers with polarographic oxygen sensors for simultaneous parallel measurements. Concentration of reagents used: 5 mM potassium glutamate, 5 mM potassium malate, 0.2 mM ADP, 50 µM 2,4-dinitrophenol (DNP). Respiratory State Definitions: Key parameters were defined as follows [14]:

V_4(0)_, basal substrate respiration;
V_3_, active respiration following ADP addition;
V_4_, metabolic state upon depletion of all ADP;
V_DNP_ (state of uncoupled respiration), maximum uncoupled respiration;
V_olig_, respiration in the presence of oligomycin;
tph, the time required for ADP phosphorylation.

The rates of oxygen consumption by mitochondria were expressed as nmol O_2_/min·mg.

### 2.8. Measurement of Mitochondrial Membrane Potential

Mitochondrial membrane potential (ΔΨm) was estimated by the distribution of tetraphenylphosphonium (TPP^+^) in the mitochondrial suspension, which was measured with a TPP^+^-sensitive electrode (Nico-Analyt, Russia). The incubation medium contained 120 mM KCl, 0.5 mM EGTA, 3 mM KH_2_PO_4_, 10 mM Hepes/KOH buffer, pH 7.4 and 5 mM glutamate + 5 mM malate. TPP^+^ was added to the incubation medium at a concentration of 1 μM. The concentration of mitochondrial protein was 0.5 mg/mL.

### 2.9. Registration of Ca^2+^ Retention Capacity by Mitochondria

The amount of Ca^2+^ necessary for the opening of the permeability transition pore (PTP) was determined using a Ca^2+^-selective microelectrode (NicoAnalyt, Moscow, Russia) after the massive loading of rat liver mitochondria with calcium. Measurements were carried out in a thermostatted cuvette under continuous stirring at 26 °C. Liver mitochondria (1.0 mg of protein) were incubated in a buffer that included 120 mM KCl, 3 mM KH_2_PO_4_, 10 mM Hepes/KOH buffer, pH 7.4 and 5 mM glutamate + 5 mM malate. Then, 15 μM Ca^2+^ was added to the reaction medium every 60 s. After several additions, the concentration of the external Ca^2+^ increased, indicating a massive release of the ion from organelles due to the opening of the PTP in the mitochondrial membrane. The calcium retention capacity (CRC) was defined as the total amount of Ca^2+^ (in nmol/mg mitochondrial protein) required to induce PTP opening.

### 2.10. Cardiolipin Assay

The cardiolipin (CL) ELISA kit (E01C2771, Blue Gene Biotech., Zhoupu Town, Pudong New Area, Shanghai, China) was used to determine the level of cardiolipin. The method is based on a competitive enzyme immunoassay using CL antigen and anti-CL-HRP conjugate. The color intensity is measured spectrophotometrically at 450 nm using a Spark Tecan plate reader (Männedorf, Switzerland).

### 2.11. Statistical Analysis

All data obtained were analyzed using GraphPad Prism 8 and Excel software and presented as mean values ± standard error of the mean. To assess the reliability of the results, a two-tailed Student’s *t*-test was used for normally distributed data or a Mann–Whitney test. Differences were considered statistically significant at *p* < 0.05.

## 3. Results

### 3.1. Assessment of the Hyperthyroidism Model

Measurement of thyroid hormone concentrations in experimental animals showed that T_3_ and T_4_ levels were increased by an average of 1.8–3.6 times. Based on this, it can be concluded that HT did develop in the experimental animals (Table 3). Due to the intensification of catabolic processes in HT, there was a decrease in total body weight, liver weight, and body weight gain (Table 3).

Administration of T_4_ resulted in an increase in the level of active carbonyl groups in serum and liver tissue by almost 50% in HRs, which may indicate an increase in the oxidative modification of biomolecules. Alanine aminotransferase (ALT) activity increased 1.6-fold, and aspartate aminotransferase (AST) activity increased 1.9-fold, while lactate dehydrogenase (LDH) activity did not change in HRs (Table 3). The data obtained indicate a differentiated effect of thyroid hormones on liver metabolism, as well as possible moderate hepatotoxicity in this HT model.

### 3.2. Analysis of Genes Responsible for Mitochondrial Cristae Biogenesis in a Rat Model of Hyperthyroidism

**Figure 1:**
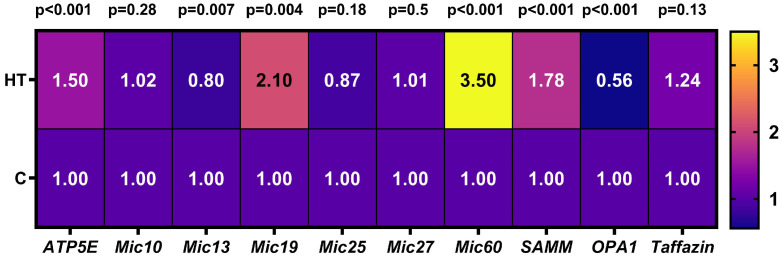
Effect of T_4_ on the baseline expression of the *MICOS* and *taffazin* genes in rat liver. A heat map displays fold change in gene expression in hyperthyroid rats compared to control rats. Fold change (2^−ΔΔCt^) is the normalized gene expression (2^−ΔCt^) in the HT group divided the normalized gene expression (2^−ΔCt^) in the control group. C, control rats; HT, hyperthyroid rats, *n* = 6–7 for C, *n* = 7 for HT.

Since the effects of thyroid hormones can be mediated through modulation of gene transcription, studies were conducted to investigate the influence of increased T_4_ dose on *MICOS* gene expression (Figure 1, Supplementary Table S1).

Expression of the *Mic10*, *Mic25*, *Mic27* and *taffazin* genes did not change in rats with hyperthyroidism. The levels of *ATP5E*, *Mic19*, *Mic60*, and *SAMM50* mRNA in HRs were increased by 1.5, 2.1, 3.5, and 1.78 times, respectively. Expression of the *OPA1* gene in HRs was reduced by almost 2 times compared to the control. This study found a small but significant decrease in *Mic13* gene expression (Figure 1).

### 3.3. Changes in MICOS Proteins in the Liver of Rats in a Hyperthyroidism Model

**Figure 2:**
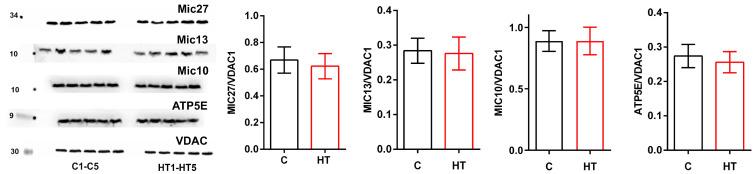
Western blot of Mic27, Mic13, Mic10, ATP5E and VDAC; C1–C5, control rats; HT1–HT5, hyperthyroid rats. Relative levels of appropriate proteins to VDAC (*n* = 5 in each group).

It was found that the levels of MIC27, 13, 10, and ATP5E proteins in mitochondria did not change at the injection of thyroxine to animals (Figure 2).

**Figure 3:**
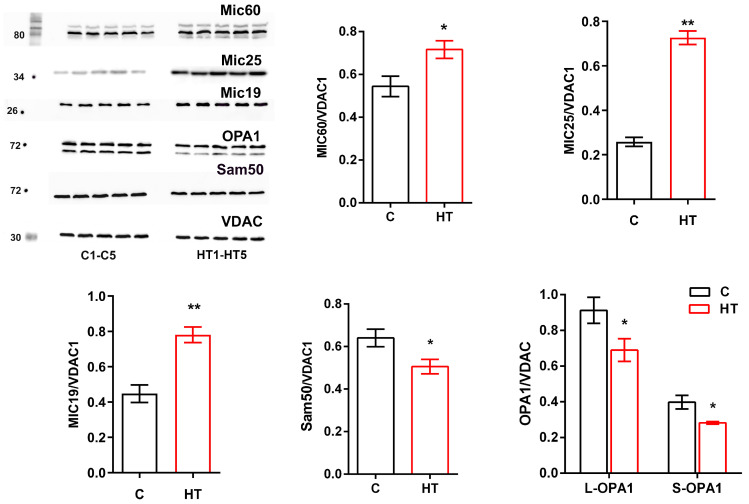
Western blot of Mic60, Mic25, Mic19, Sam50, OPA1 and VDAC; C1–C5, control rats; HT1–HT5, hyperthyroid rats. Relative levels of appropriate proteins to VDAC. * *p* < 0.05; ** *p* < 0.02 as compared with the control data (*n* = 5 in each group).

The excess of T_4_ affected the level of other MICOS proteins: there was a 32% increase in MIC60, a 190% increase in MIC25, and a 74% increase in MIC19. At the same time, the levels of Sam50, as well as the long and short forms of OPA1, were reduced by 20 to 30% in the mitochondria liver of HRs (Figure 3).

Thus, it can be concluded that a high dose of thyroxine alters expression of genes and synthesis of MICOS proteins.

### 3.4. Hyperthyroidism—Induced Changes in MICOS Balance Affect the Ultrastructure of Mitochondria in the Liver of Hyperthyroid Rats

**Figure 4:**
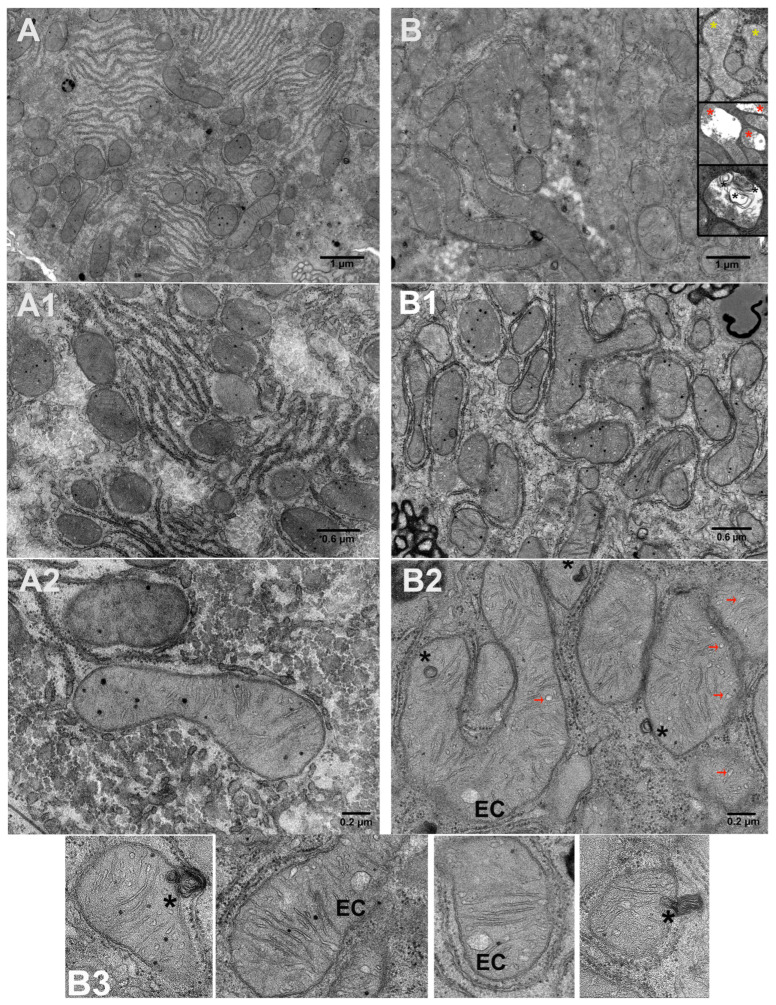
TEM images of hepatic tissue in the control (**A**–**A2**) and in experimental hyperthyroidism (**B**–**B3**). The insets in (**B**) represent examples of mitochondria found in hepatocytes of hyperthyroid rats. The number of examined images in each group was about 40. Black asterisks, multilamellar bodies; yellow asterisks, swollen mitochondria; red asterisks, damaged mitochondria; EC and red arrows, expanded mitochondrial cristae.

The morphology of mitochondria (shape, size, ultrastructure) can change in response to the energy needs of the cell, oxidative stress, or pathological conditions. The modifications in the level of MICOS proteins led to the remodeling of the structure of liver mitochondria in HRs (Figure 4, Table 4).

In the control group, mitochondria mainly had well-defined membrane boundaries and tightly packed cristae, as well as an electron-dense matrix. In CR hepatocytes, oval or rounded mitochondria were typically found, while HRs exhibited organelles with worm-like, branched, highly elongated, or spherical shapes. This change in organelle shape manifested itself in the elongation of the major axis relative to the minor axis (the so-called aspect ratio) (Table 4). Ultrastructural analysis of liver tissue revealed noticeable changes in the HR mitochondrial population. The samples contained swollen organelles with enlarged intercrista space (14%) (Figure 4B, insets in Figure 4B, Table 4). Completely damaged organelles were also found, with complete or partial vacuolization of the matrix, membrane destruction, and reduced number of cristae (11.3%) (Figure 4B, inserts; Table 4). Almost every HR mitochondrion was surrounded by a rough endoplasmic reticulum. Large lipid droplets were frequently found in HR liver cells (Figure 4(B1), upper right corner). HT caused an increase in the number, area, and perimeter of organelles in rat livers. The individual megamitochondria in hyperthyroid animals had an area greater than 1–1.5 µm^2^ and a perimeter greater than 6 µm. An increased concentration of T_4_ caused a 22% decrease in the number of cristae relative to the cross-section of mitochondria, as well as a 12% increase in the width of regular cristae (Table 4). In some organelles, multilamellar bodies (concentric layers of membrane) (MLB) were found, located either at the edge of or inside the organelles. Apparently, some peripheral MLB were released from mitochondria (Figure 4(B2) and insets in Figure 4B).

**Figure 5:**
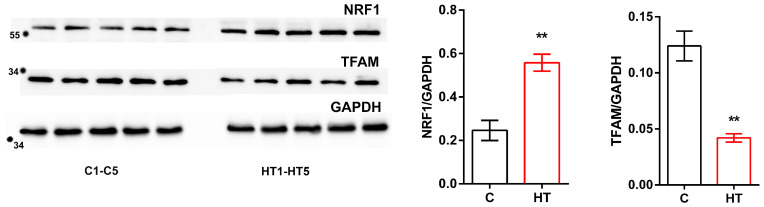
Western blot of NRF1, TFAM and GAPDH; C1–C5, control rats; HT1–HT5, hyperthyroid rats. Relative levels of appropriate proteins to GAPDH. ** *p* < 0.02 as compared with the control data (*n* = 5 in each group).

We have previously demonstrated the stimulation of PGC-1a, a member of the transcription coactivator family that mediates mitochondrial biogenesis in HR liver [5]. This work also revealed that HT caused an increase in the level of nuclear respiratory factor 1 (NRF1), but led to a decrease in mitochondrial transcription factor A (TFAM) (Figure 5).

### 3.5. Effect of MICOS Protein Changes in Hyperthyroidism on the Functional State of Liver Mitochondria

Thyroid hormones regulate the metabolic rate, so mitochondria can be considered the main cellular site where metabolic transformations occur. Abnormal cristae morphology is associated with mitochondrial dysfunction and is considered a sign of many human pathologies, including obesity and diabetes mellitus, cardiomyopathy, muscular dystrophy and myopathy, as well as neurodegenerative conditions such as Alzheimer’s and Parkinson’s diseases [15,16,17]. This study assessed the functional state of liver mitochondria by measuring respiration and oxidative phosphorylation parameters using NAD-dependent substrates (Table 5).

**Figure 6:**
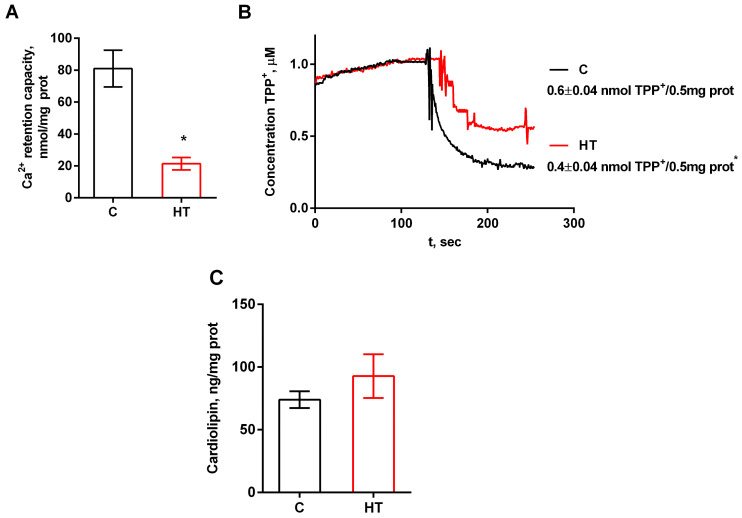
Calcium retention capacity (**A**), membrane potential (**B**) and cardiolipin level (**C**) in isolated liver mitochondria of control and hyperthyroid rats. C, control rats; HT, hyperthyroid rats. * *p* < 0.05 as compared with the control data (*n* = 5 in each group).

HR liver mitochondria demonstrate increased oxygen consumption in almost all metabolic states except for uncoupled respiration (Table 5). Increased values of V_4_ and V_olig_ indicate elevated proton leakage through the membrane, which is characteristic of HT. The V_DNP_ parameter did not change in this case, i.e., the maximum electron transport rate in the mitochondrial respiratory chain in hyperthyroid animals was preserved. We revealed a 23% decrease in the respiratory control ratio in HR liver mitochondria. The functional efficiency of mitochondria can be influenced by parameters such as Ca^2+^ transport and changes in membrane potential. The higher the calcium retention capacity, the more resistant mitochondria are to Ca^2+^-induced PTP opening, which is important for their functional integrity. As shown in Figure 6A, the ability of mitochondria to retain calcium ions in liver mitochondria was reduced by 3.8 times in HRs (22 ± 4 µM Ca^2+^) compared to CRs (81 ± 12 µM Ca^2+^) (Figure 6A).

In addition, in animals with hyperthyroidism, the mitochondrial membrane potential was reduced by ≈33% (Figure 6B). This may indicate a dysfunction of the electron transport chain and, accordingly, lead to a decrease in proton gradient generation. The concentration of cardiolipin was shown not to change significantly in HRs (Figure 6C). The values of all the above parameters apparently depend on the maintenance of the normal structure of mitochondrial membranes. Thus, we propose that changes in the MICOS complex underlie the mitochondrial functional impairment in rats with HT, which is manifested as a hypermetabolic effect, compromised phosphorylation efficiency, and reduced CRC in liver mitochondria.

## 4. Discussion

Mitochondrial energetic adaptation encompasses a variety of conserved processes that support the adaptability and survival of cells and organisms in changing environments by regulating mitochondrial respiratory capacity. Some of the key regulatory factors of energetic adaptation are mitochondrial biogenesis and membrane dynamics. Failure or reduction in the adaptive remodeling of cristae causes cell damage, inflammation, or aging, which compromises cell survival and the ability to adapt to new environmental conditions with increased energy demands [7]. The structural unit that ensures the correct spatial distribution and functioning of the respiratory complexes is the crista. Cristae can dynamically change [18] to adapt to altering energy requirements and physiological signals [19,20,21,22]. As already mentioned, cristae are connected to the inner limiting membrane by small pores called crista junctions (CJs) [23]. CJs, being 12–40 nm in diameter, potentially regulate many mitochondrial functions: they optimize electron transfer and reduce the production of reactive oxygen species, maintain local acidification in the cristae, which is necessary for the efficient functioning of ATP synthase, and participate in apoptosis. Cristae organization requires an optimal combination of the MICOS complex, OPA1, F_1_Fo-ATP synthase, and the correct ratio of cardiolipin and phosphatidylethanolamine. MICOS is an evolutionarily conserved multi-subunit protein ensemble that is anchored at the sites of CJs. MICOS consists of the following proteins: Mic10/Minos1, Mic26/APOO, Mic27/APOOL, Mic13/QIL1, Mic19/CHCHD3, Mic25/CHCHD6, and Mic60/MITOFILIN/IMMT [24]. The above proteins are organized into two subcomplexes: the Mic60 subcomplex (Mic60–Mic19–Mic25) and the Mic10 subcomplex (Mic13–Mic10–Mic26–Mic27) [25]. The concerted function of these heterogeneous subunits is a prerequisite for the complex’s collective activity in mediating the formation and maintenance of cristae architecture [9]. The loss of any single MICOS subunit disrupts cristae junction integrity, leading to their disappearance or reduction, detachment of cristae from IBM, and structural deformations [26].

In this work, we investigated the interaction of/changes in the main components of cristae formation and how these changes affect the functional state of liver mitochondria in rats with experimentally induced hyperthyroidism. To confirm the induced model of hyperthyroidism, a standard method for determining the concentration of free forms of T_3_ and T_4_ in blood serum was used (Table 3). In HRs, the concentration of these hormones was significantly increased, indicating a state of HT. In addition, a decrease in body and liver weight, and body weight gain in rats further confirmed the development of pathology. Unchanged LDH activity in the serum of the investigated rats could be a consequence of accelerated aerobic metabolism [27] in the absence of significant hemolysis or tissue necrosis [28] (Table 3). The observed increase in AST and ALT activities was probably a consequence of increased catabolism of muscle proteins and oxidative stress [4,29]. We have previously shown that in HT the rate of H_2_O_2_ formation increases at an unchanged glutathione peroxidase activity in the liver [4], and the observed increase in active carbonyl groups in the HR blood serum may be one of the early indicators of tissue damage in free radical pathology (Table 3).

Analysis of *MICOS* gene expression and protein levels showed that the Mic10 subcomplex did not change in HR liver, with the exception of a small but significant decrease in the expression of the *Mic13* gene, whose protein binds the Mic60 and Mic10 subcomplexes, which may not be a critical indicator in this case (Figure 1 and Figure 2). Administration of T_4_ caused a significant increase in both *Mic60* gene expression and Mic60 levels. Given that Mic60 is necessary for the formation of CJs and contact sites between the IM and OM, its sharp activation indicates a serious restructuring of the mitochondrial contact system. The presented study also revealed a nearly twofold increase in *Mic19* mRNA and Mic19 protein levels and a threefold increase in Mic25 protein (Figure 1 and Figure 3). Mic19 and Mic25 play crucial roles in the assembly and stability of the MICOS complex [30,31]. Thus, it can be said that hyperthyroidism is accompanied by a disproportion of the MICOS: the Mic60 subcomplex is activated, while the Mic10 subcomplex remains unchanged. Nevertheless, the observed stability of subcomplex 10 ensures the preservation of the basic cristae architecture at the level of curve formation. Interestingly, despite the increased expression of the *SAMM50* gene, the level of this protein decreased by 20% in HRs (Figure 1 and Figure 3). Sam50 is one of the major proteins of the Sorting and Assembly Machinery (SAM) system, which interacts with the MICOS and regulates cristae morphology by participating in the creation of contact sites between two mitochondrial membranes [26]. With a decrease in Sam50 levels, the number of CJs also decreases [32,33], suggesting that contact sites stabilize CJs, possibly acting as an anchor [34]. Since Sam50 is associated with MICOS via the Mic60–Mic19–Sam50 axis [33], increased expression of the *Mic19* and *Mic60* genes and their proteins may be a compensatory mechanism in maintaining the stability of CJs due to a decrease in the content of Sam50. Taking into account the above, it can be concluded that Sam50 may be one of the main targets in this HT model. The discrepancy between gene expression and protein levels can be explained by many reasons, such as a more stable state of mRNA under the influence of thyroid hormones or post-translational modifications that can cause inhibition of protein synthesis despite increased gene expression. Changes in the expression/levels of proteins within the MICOS complex are observed in various pathologies. Overexpression of Mic60 leads to increased CJs formation, branching of cristae, enlargement of CJs diameter, and reduced levels of F_1_F_O_ supercomplexes [35]. In experiments with artificially induced overexpression of MIC60, cardiac hypertrophy was also observed, with its degree exceeding the hypertrophy caused by other external factors [36]. An imbalance in the levels of Mic60 and Mic19 is associated with various types of cancer [37,38]. Overexpression of Mic25 increases cell resistance to the anticancer drugs etoposide and doxorubin, while the loss of Mic25 increases their sensitivity to these drugs [30]. Interestingly, despite the observed cristae abnormalities in non-alcoholic fatty liver disease, levels of Mic60, Mic25, Mic19, and Sam50 are increased in the liver of mice fed a high-fat diet [39,40,41].

Figure 1 shows that the expression of the gene encoding ATP5E, another component necessary for cristae organization, increased 1.5-fold, while the protein level itself remained unchanged. ATP5E imparts positive curvature to membranes, which minimizes the presence of cristae arranged in concentric circles and resembling an onion-like structure [42,43]. Another protein involved in cristae biogenesis is OPA1, which is another target of thyroid hormones. We found that HT significantly reduced both *OPA1* gene expression and L- and S-OPA1 levels (Figure 1 and Figure 3). OPA1 regulation is complex, as it involves 8 alternative splicing variants and two proteolytic cleavage sites that generate multiple forms of OPA1 (membrane-bound L-OPA1 and soluble S-OPA1). OPA1 promotes oligomerization of F_1_F_O_-ATP synthase, protecting mitochondria from respiratory stress [44]. The role of OPA1 as a regulator of cristae is emphasized by studies showing that cells lacking OPA1 not only exhibit highly fragmented mitochondria but also exhibit excessively dilated and dysfunctional cristae, mitochondrial dysfunction, and increased susceptibility to cell death stimuli [45,46]. This is consistent with our data showing the presence of abnormal and enlarged cristae in HR liver mitochondria compared to control (Figure 4 and Table 4). However, OPA1 reduction results in only a modest change in mitochondrial phenotype compared to Mic10 or Mic60 knockouts [47], and CJs can also form in the absence of OPA1 [48]. Perhaps in our case, overexpression of MIC60 is also compensatorily induced by a decrease in OPA1 level, which is consistent with the data of Barrera M. et al. [48]. Decreased or absent taffazin, a mitochondrial acyltransferase that converts cardiolipin to its mature form, causes an abnormal enlargement of cristae arranged in concentric stacks or highly interconnected cristae in cardiac muscle [49]. In this study, we found no changes in either *taffazin* gene expression or cardiolipin concentrations in HR liver (Figure 1 and Figure 6C).

Disruption of the normal balance of components involved in cristae organization also caused noticeable visual changes in the ultrastructure of HR liver mitochondria (Figure 4 and Table 4). HR hepatocyte preparations contained swollen and damaged mitochondria; organelles were represented by a variety of shapes, unlike the usual oval or round organelles. HT caused increased biogenesis and affected mitochondrial dynamics in the livers of experimental animals. As for the presence of multilamellar bodies in organelles (Figure 4, inserts), since PINK1/Parkin-dependent mitophagy (Supplementary Figure S1) in HR mitochondria is impaired, this is, probably, compensated for by the fact that mitochondrial membrane material can be used to form the developing autophagosome in this pathology (Figure 4(B3)) [50]. Interestingly, T_4_ had a multidirectional effect on key regulators of mitochondrial biogenesis. An increase in the level of nuclear respiratory factor 1 (NRF1) is consistent with the previously demonstrated activation of PGC-1α, indicating the activation of a nuclear genetic program aimed at increasing the number of mitochondria in response to increased energy demands caused by excess thyroid hormones [5,51]. However, despite activation of the upstream regulator NRF1, the level of TFAM protein, which is the final effector directly responsible for mitochondrial DNA replication and transcription, reduced in hyperthyroidism (Figure 5). Our data are consistent with the results of Bonekamp et al., who showed that the TFAM-to-mtDNA ratio is critical for maintaining normal mitochondrial gene expression, and excessive TFAM levels can lead to transcriptional silencing rather than activation [52].

Structural changes in MICOS are directly linked to mitochondrial functional activity. Functional assessment of liver mitochondria of HRs showed increased mitochondrial respiration in states V_3_ and V_4_, but a decreased respiratory control ratio (Table 3). The decrease in both the RCR and the membrane potential (Figure 6B) indicates impaired coupling efficiency between respiration and ATP synthesis, as well as altered integrity of mitochondria in HRs. Mitochondria of HRs are characterized by increased proton leakage, which persists even after blocking ATP synthase with oligomycin (V_olig_). Although the ability to oxidize substrates is preserved in HR mitochondria, the observed uncoupling of oxidative phosphorylation leads to increased heat production, which underlies hypermetabolism. However, the maximum electron transport chain rate in the mitochondria of hyperthyroid animals remained unchanged. As the MICOS complex interacts with multiple proteins in various mitochondrial compartments, it is logical to assume that these interactions have important physiological significance. Recently, MICU1 hexamers were found to interact with mitochondrial calcium uniporter (MCU) in the IM and control cristae width independently of their function in calcium signaling. MICU1 depletion results in an increase in CJ diameter, increased cytochrome c release, and a loss of membrane potential [53]. As Figure 6A shows, there was a significant decrease in CRC (3.8-fold), indicating impaired Ca^2+^ buffering function and a high probability of mPTP opening (Figure 6A). The mPTP is a mega-channel consisting of proteins from both the inner and outer mitochondrial membranes. Any changes in the composition or level of pore proteins, membrane potential, disruption of the cardiolipin/phosphatidylethanolamine ratio, and an increase in the production of reactive oxygen species are critical for normal functioning of the pore. We have previously demonstrated an increase in the levels of the channel-forming subunit MCU and cyclophilin D in an experimental model of hyperthyroidism. The level of the regulatory MICU1 in that case remained virtually unchanged [54]. An increase in the adenine nucleotide translocator ANT2 in HT, another mPTP component, could also affect the properties of mPTP (Supplementary Figure S2). Thus, structural rearrangements in the MICOS complex directly influence functional changes in liver mitochondria of rats with hyperthyroidism.

## 5. Conclusions

**Figure 7:**
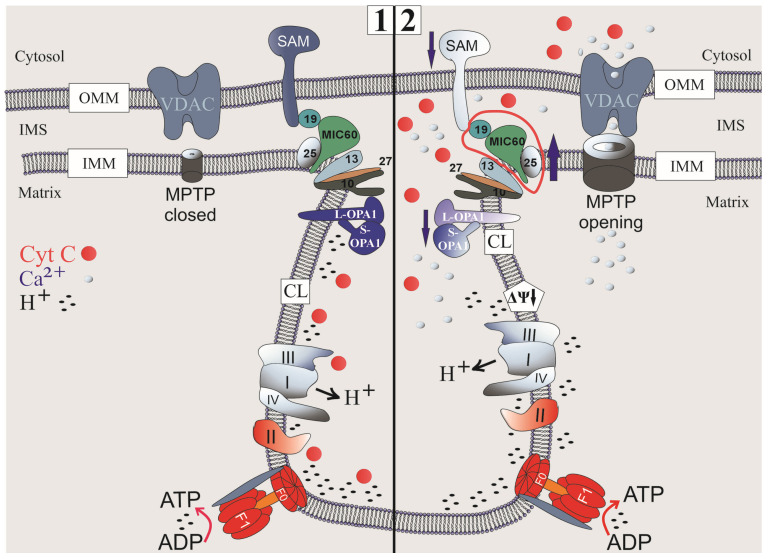
Components of mitochondrial cristae in normal conditions (**1**) and in HT (**2**).

In hyperthyroidism, there is a significant decrease in the level of OPA1 and Sam50 and selective hyperactivation of the MIC60 subcomplex while maintaining the stability of the MIC10 subcomplex, which creates a functional imbalance, disrupts cristae architecture, and critically impacts energy metabolism and Ca^2+^ homeostasis in the liver mitochondria of animals with this pathology (Figure 7). This may largely explain the high incidence of liver complications in severe hyperthyroidism and emphasizes the importance of early correction of thyroid status to prevent irreversible damage to liver mitochondria [55,56]. It can be argued that the development of methods for restoring the balance of proteins involved in the formation of cristae and CJs, such as MICOS, OPA1, and SAM50 in hyperthyroidism, represents a new direction in the treatment of hyperthyroidism and other metabolic disorders. A clear example of this was the demonstration of successful restoration of mitochondrial function when the OPA1 protein deficiency was eliminated in Autosomal Dominant Optic Atrophy [57]. At the same time, in order to establish a complete situation of the cause-and-effect relationships of disorders in hyperthyroidism, it is necessary and planned to conduct a series of additional studies using animals deficient in MICOS, OPA1, and SAM 50.

## Figures and Tables

**Table 1:** List of gene-specific primers used for RT-qPCR.

Gene	Forward (5′ → 3′)	Reverse (5′ → 3′)
*Micos10*	GACGCGTTAGTGAAGCTAGG	TGCTGACAGTTGGAGTAGGC
*Atp5f1e*	ACAGGCTGGACTCAGCTAC	TCTCAGCGTTCGCTTTGAAC
*Micos13*	AGCAATGTACCAGTTCAGCC	GACCGAGATGATGCCTGAGT
*Micos 19 (Chchd3)*	CAAAGGTGAAGCATCTGGCTC	CATACCGCCTGAACTTGGAC
*Micos 25 (Chchd6)*	GTCAGATCGCCTAACCAGGG	TCTCTTGGATGCGTTCCTGC
*Micos 27 (Apool)*	CCAAAGGAAGAAACCAAGGAAGG	CATGAGCTTGGGGTCAGGTAT
*Micos 60 (Immt)*	TGGTCCAAGCAAGGGATGAC	AGTGGAGAGTGTGCCAGCT
*Samm50*	GCCTTGCTCAAAGTCAACCA	GACGTGGAGAACACCGAATC
*OPA1*	GCAGAAGACAGCTTGAGGGT	TGCGTCCCACTGTTGCTTAT
*Tafazzin*	GCGCTTCAAATGGGGAATTG	GTAGGGTGGACTGTTAGGGA
*Rplp2*	CATCGCTCAGGGTGTTGG	AACAGGCCAAATCCCATGTC
*PPIA*	GAGCACTGGGGAGAAAGGATT	GTTTGGTCCAGCATTTGCCA

**Table 2:** Antibodies used for Western blot analysis.

Antibodies	Source
TFAM	AF0531, Affinity Biosciences, Zhenjiang, China
NRF1	AF5298, Affinity Biosciences
OPA1	ab119685, Abcam, Cambridge, UK
Mic 60 (Mitofilin)	DF7074, Affinity Biosciences
Samm 50	DF12729, Affinity Biosciences
Mic 27 (ApoOL)	AF9010, Affinity Biosciences
Mic 25 (CHCHD6)	DF12255, Affinity Biosciences
Mic 19 (CHCHD3)	DF12254, Affinity Biosciences
Mic 10 (MINOS1)	DF14761, Affinity Biosciences
ATP5E	DF9238, Affinity Biosciences
Mic 13 (Qil1)	PA5-69966, Invitrogen, Burlington, ON, Canada
GAPDH	ab181602, Abcam, Cambridge, UK
VDAC	ab154856, Abcam, Cambridge, UK

**Table 3:** Somatic and biochemical characteristics of control and hyperthyroid rats.

	C	HT
T_3 free_, pmol/L	6.5 ± 0.3	12.8 ± 0.8 **
T_4 free_, pmol/L	27.5 ± 1.3	78.5 ± 1.5 **
Body weight, g	263 ± 3	243 ± 3 **
Liver weight, g	11.8 ± 0.2	9.6 ± 0.2 **
Body weight gain, g	39 ± 1.8	12 ± 1.1 **
ALT, µmol/min·L	34.2 ± 1.6	54.8 ± 3.3 **
AST, µmol/min·L	57.5 ± 2.3	111.4 ± 5.4 **
LDH, µmol/min·L	374 ± 12	389 ± 16
Total carbonyl, serum, µg carbonyl group/mg protein	4.7 ± 0.3	7 ± 0.6 *
Total carbonyl, tissue, µg carbonyl group/mg protein	9.9 ± 0.6	14.3 ± 0.7 *

* *p* < 0.05; ** *p* < 0.02 as compared with the control data (*n* = 10 in each group). C, control rats; HT, hyperthyroid rats; MX, mitochondria.

**Table 4:** Morphometric parameters of rat liver mitochondria of control and hyperthyroid rats.

	C	HT
Number of mitochondria per image (60 µm^2^)	27 ± 0.8	40 ± 1.2 *
Percentage of swollen mitochondria	2	14
Percentage of mitochondrial damage	3	11.3
Average area of mitochondria, µm^2^	0.37 ± 0.01	0.50 ± 0.02 **
Average perimeter of mitochondria, µm	2.30 ± 0.04	2.80 ± 0.05 **
Aspect ratio (major axis/minor axis)	1.7 ± 0.04	2.1 ± 0.05 **
Number of lamellar regular cristae/area MX	215.1 ± 20.5	168.4 ± 16 **
Width of regular lamellar cristae, nm	14.6 ± 0.54	16.5 ± 0.28 *

For morphometry, 40 to 50 images per animal were analyzed. C, control rats; HT, hyperthyroid rats. * *p* < 0.05; ** *p* < 0.02 as compared with the control data (*n* = 3 in each group).

**Table 5:** Respiration parameters and oxidative phosphorylation of liver mitochondria of control and hyperthyroid rats using glutamate and malate.

	C	HT
V_4(0)_	3.9 ± 0.3	5.2 ± 0.6 *
V_3_	41.8 ± 1.8	52.8 ± 3.1 *
V_4_	4.4 ± 0.2	7.2± 0.3 *
V_olig_	4.0± 0.1	5.2± 0.3 *
V_DNP_	51.7± 1.3	47.9± 2.9
RCR (V_3_/V_4_)	9.5± 0.3	7.3± 0.2 *
tph, s	0.87± 0.03	0.81± 0.04

V_4(0)_, V_3_, V_4_, V_DNP_, rate of respiration in different metabolic states, nmol O_2_/min·mg of protein; V_olig_, rate of respiration in the presence of oligomycin; tph, ADP phosphorylation, s. Additions: 5 mM glutamate + 5 malate; 0.2 mM ADP; 0.05 mM DNP; mitochondrial protein, 1 mg/mL. C, control rats; HT, hyperthyroid rats. * *p* < 0.05 as compared with the control data (*n* = 10 in each group).

## Data Availability

Data are available within the article and Supplementary Materials.

## Supplementary Materials

The following supporting information can be downloaded at: https://www.mdpi.com/article/10.3390/cells14231877/s1, Figure S1: Western blot of PINK1 and Parkin; C1–C5—control rats, T1–T5—hyperthyroid rats. Relative levels of appropriate proteins to VDAC. ** *p* < 0.02 as compared with the control data; Figure S2: Western blot of ANT2; C1–C6—control rats, T1–T6—hyperthyroid rats. Relative levels of appropriate proteins to VDAC. ** *p* < 0.02 as compared with the control data; Table S1: The baseline expression of the *MICOS* and *taffazin* genes in liver of Hyperthyroid Rats vs. Control Rats.
