# Supplementary material for: Imbalance in MICOS Proteins in Rat Liver Mitochondria in an Induced Hyperthyroidism Model
Source: Cells. 2025 Nov 27;14(23):1877. doi: 10.3390/cells14231877 (PMC12691126; doi:10.3390/cells14231877)
Supplement: Supplementary file 1 [file cells-14-01877-s001.zip › cells-3978195-supplementary.pdf]

Supplementary Materials

Article: **Imbalance in MICOS proteins in rat liver mitochondria in an induced hyperthyroidism model.**

Natalya Venediktova, Ilya Solomadin, Anna Nikiforova and Bessonova Tatiana.

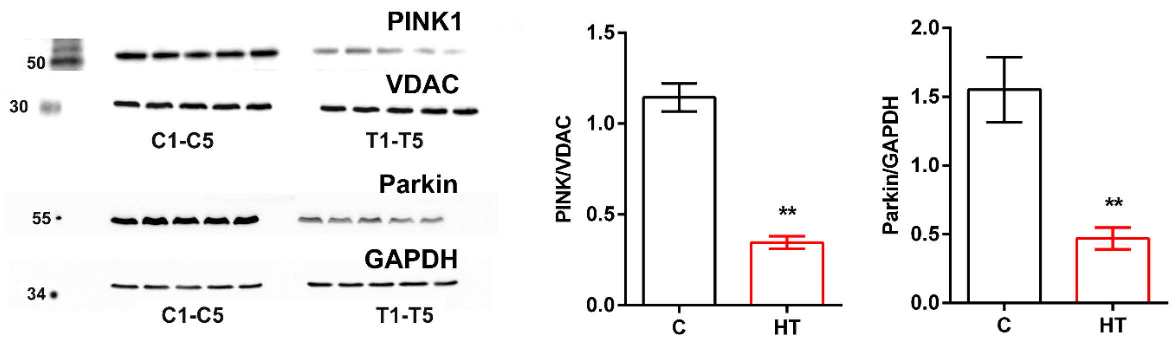

Figure S1. Western blot of PINK1 and Parkin; C1-C5 - control rats, T1-T5 - hyperthyroid rats. Relative levels of appropriate proteins to VDAC. ** $p < 0.02$ as compared with the control data.

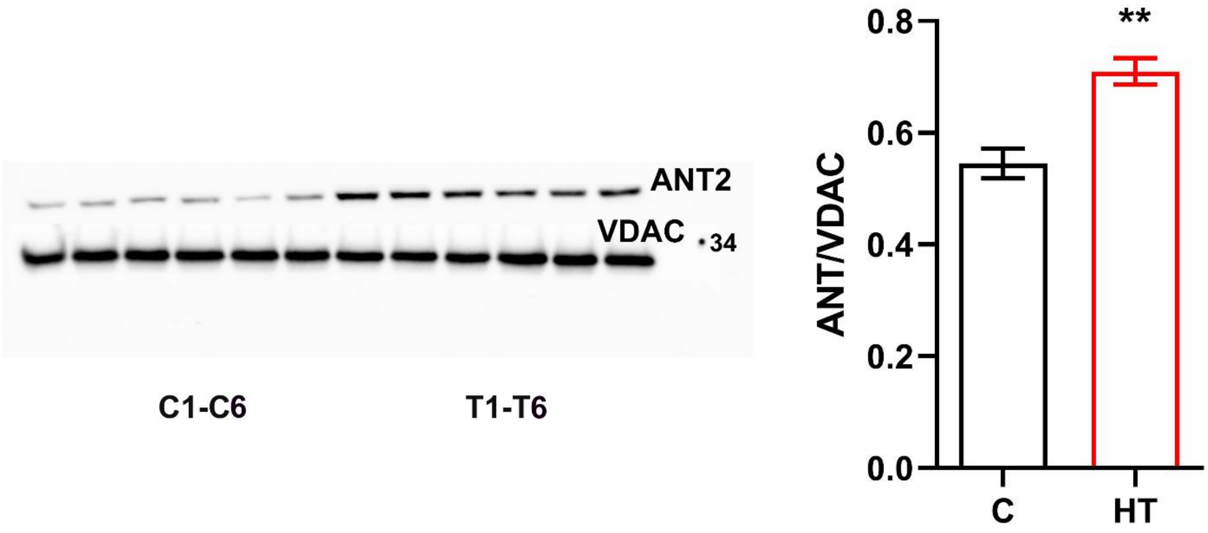

Figure S2. Western blot of ANT2; C1-C6 - control rats, T1-T6 - hyperthyroid rats. Relative levels of appropriate proteins to VDAC. ** $p < 0.02$ as compared with the control data.

Table S1. The baseline expression of the *MICOS* and *taffazin* genes in liver of Hyperthyroid Rats vs Control Rats.

	ATP5E	*Mic10*	*Mic13*	*Mic19*	*Mic25*	*Mic27*	*Mic60*	*SAMM50*	*OPA1*	*Taffazin*
**C**	1.00±0.11	1.00±0.06	1.00±0.07	1.00±0.07	1.00±0.08	1.00±0.16	1.00±0.25	1.00±0.17	1.00±0.08	1.00±0.15
**HT**	1.5±0.13**	1.02±0.14	0.80±0.17	2.10±0.35**	0.87±0.11	1.01±0.08	3.50±0.65**	1.78±0.09*	0.56±0.05**	1.24±0.13

* $p < 0.05$; ** $p < 0.02$ as compared with the control data. C, control rats; HT, hyperthyroid rats, n=6-7 for C, n=7 for HT
